# Electronic cigarette exposure triggers neutrophil inflammatory responses

**DOI:** 10.1186/s12931-016-0368-x

**Published:** 2016-05-17

**Authors:** Andrew Higham, Nicholas J. W. Rattray, Jennifer A. Dewhurst, Drupad K. Trivedi, Stephen J. Fowler, Royston Goodacre, Dave Singh

**Affiliations:** Manchester Academic Health and Science Centre, University Hospital of South Manchester Foundation Trust, Centre for Respiratory and Allergy Medicine, Institute of Inflammation and Repair, Faculty of Medical and Human Sciences, The University of Manchester, Manchester, UK; Manchester Institute of Biotechnology, School of Chemistry, The University of Manchester, Manchester, UK

**Keywords:** Electronic cigarettes, COPD, Inflammation, Smoking, Neutrophils, MMP-9

## Abstract

**Background:**

The use of electronic cigarettes (e-cigs) is increasing and there is widespread perception that e-cigs are safe. E-cigs contain harmful chemicals; more research is needed to evaluate the safety of e-cig use. Our aim was to investigate the effects of e-cigs on the inflammatory response of human neutrophils.

**Methods:**

Neutrophils were exposed to e-cig vapour extract (ECVE) and the expression of CD11b and CD66b was measured by flow cytometry and MMP-9 and CXCL8 by ELISA. We also measured the activity of neutrophil elastase (NE) and MMP-9, along with the activation of inflammatory signalling pathways. Finally we analysed the biochemical composition of ECVE by ultra-high performance liquid chromatography mass spectrometry.

**Results:**

ECVE caused an increase in the expression of CD11b and CD66b, and increased the release of MMP-9 and CXCL8. Furthermore, there was an increase in NE and MMP-9 activity and an increase in p38 MAPK activation. We also identified several harmful chemicals in ECVE, including known carcinogens.

**Conclusions:**

ECVE causes a pro-inflammatory response from human neutrophils. This raises concerns over the safety of e-cig use.

**Electronic supplementary material:**

The online version of this article (doi:10.1186/s12931-016-0368-x) contains supplementary material, which is available to authorized users.

## Background

There are an estimated 13 million users of electronic cigarettes (e-cigs) worldwide [1]. E-cigs are used to help reduce or stop tobacco smoking [2]. However, it has been shown that toxic chemicals are present in e-cig vapour, such as formaldehyde and acrolein [3–5], casting doubt on the safety of using e-cigs.

Cigarette smoke extract increases the secretion of pro-inflammatory mediators from a range of different cell types including epithelial cells, macrophages and neutrophils [6–8]. Similarly, e-cig vapour exposure increases the release of inflammatory mediators from keratinocyte and alveolar epithelial cell lines [9]. Furthermore, e-liquid increases interleukin-6 secretion from bronchial epithelial cells [10]. This raises concerns over the potential of e-cigs to promote pulmonary inflammation in a similar manner to tobacco smoking.

Neutrophil numbers are increased in the lungs of chronic obstructive pulmonary disease (COPD) patients and increased numbers positively correlate with disease severity [11, 12]. Chemokine C-X-C motif ligand 8 (CXCL8) is a key neutrophil chemoattractant [13] and the levels of CXCL8 are increased in the lungs of COPD patients [14]. Cigarette smoke exposed neutrophils secrete CXCL8, which may lead to increased neutrophil recruitment to the lungs.

Neutrophils are involved in many aspects of COPD pathophysiology. For example, neutrophils release proteases such as neutrophil elastase (NE) and matrix metalloproteinase-9 (MMP-9) which cause tissue destruction resulting in emphysema [15]. Cigarette smoke stimulates NE and MMP-9 release from neutrophils [8, 16]. The levels of NE and MMP-9 are increased in the airways of COPD patients and the levels positively correlate with disease severity [17–19]. Moreover, COPD neutrophils demonstrate a higher level of activation compared to controls {Wright, 2016 #132}.

Upon exposure to cigarette smoke and the bacterial peptide N-formylmethionyl-leucyl-phenalanine (fMLP), neutrophils increase the expression of CD11b and CD66b, important for the migration and degranulation of neutrophils at sites of inflammation [20, 21]. There is increased expression of the adhesion molecule CD11b on the surface of blood and lung neutrophils of COPD patients [22–25]. CD11b and CD66b can be regarded as markers of neutrophil activation.

The effects of e-cigs on neutrophil activity are unknown. Due to the complex mixture of chemicals present in e-cigs, we hypothesised that exposure to e-cigs will induce neutrophil activation. The aim of this study was to investigate the effects of e-cig exposure on the inflammatory response of human neutrophils. We examined the effects on CD11b, CD66b and neutrophil shape change, and the release of MMP-9, NE and CXCL8, which are all implicated in the pathogenesis of COPD [17, 26, 27].

## Methods

### Study subjects

Ten healthy never-smokers were recruited to donate peripheral blood samples (demography shown in Additional file 1). All subjects gave written informed consent. This research was approved by Preston research ethics committee REC reference 10/H1016/25.

### Electronic cigarette

Table 1 shows the three brands of e-cigs used for this study; VIP® (brand 1), KIK® (brand 2) and Puritane® (brand 3). All experiments were performed using brand 1 containing 24 mg nicotine unless otherwise stated.

**Table 1:** Brands of e-cig used in the study

Brand	VIP (brand 1)		KIK (brand 2)		Puritane (brand 3)
Model	1100mAh battery with V5/CE5 clearomiser		900mAh battery with protank clearomiser		Re-chargeable battery with replacement cartomiser
Flavour	USA Tobacco		USA Red		Original
Nicotine Strength	0 mg	24 mg	0 mg	24 mg	16 mg

A summary of the brands, models of of e-cig and flavour of e-liquid used to generate e-cig vapour extract preparations for this study

### E-cig vapour extract preparation

For brand 1 and brand 2, e-cig vapour extract (ECVE) was prepared by bubbling e-cig vapour through RPMI-1640 culture medium (Sigma-Aldrich, Poole, UK) supplemented with 2 mM L-glutamine (Invitrogen, Paisley, UK), 100 U/mL penicillin and 100 μg/mL streptomycin (Sigma-Aldrich) using a Watson-Marlow 520R peristaltic pump (Watson-Marlow Ltd, Falmouth, UK). Vapour was generated by manual activation of the heating coil at a flow rate of 15 mL/min. For brand 3, vapour was generated automatically using a flow rate of 90 mL/min. Each cycle of vapour lasted 10 s followed by a 30 s interval. In total for each brand, e-cig vapour was bubbled through the media 20 times and the total time of preparation was approximately 13 mins.

For all experiments, ECVE optical density (OD) was measured using a spectrophotometer (Eppendorf, Stevenage, UK)) at the 320 nm wavelength, as is the case for previous publications using cigarette smoke extract [7, 28, 29]. Optical density is an arbitrary unit used to find the concentration of ECVE which gives the optimal response and to normalise preparations for the purposes of obtaining reproducible data. The choice of 320 nm wavelength was based upon the relative contribution of this peak in all of the ECVE preparations (Additional file 2) determined using a POLARstar Omega microplate reader (BMG Labtech, Buckinghamshire, UK); this was a major peak in the absorbance spectrum for all ECVE preparations. It was important to avoid peaks at 260 nm for two reasons; 1) the absorbance spectra of nicotine demonstrate a major peak at 260 nm [30, 31]; 2) The quantification of nucleic acids utilises absorbance at 260 nm. Any contaminating nucleic acids may therefore affect the results at this part of the spectrum. Foetal calf serum (FCS; 10 %, Invitrogen) was then added and the ECVE was filtered using a syringe driven 0.22 μm filter. The ECVE was then adjusted to the desired OD using culture medium.

### Cigarette smoke extract preparation

Cigarette smoke extract (CSE) was prepared as previously described [7]. Briefly, one 3R4F Kentucky research cigarette (University of Kentucky, Kentucky, USA) was bubbled through RPMI-1640 culture medium supplemented with 2 mM L-glutamine, 100 U/mL penicillin and 100 μg/mL streptomycin using a Watson-Marlow 520R peristaltic pump. 10 % FCS was then added and the CSE was filtered using a syringe driven 0.22 μm filter. The CSE was then adjusted to the desired OD using culture medium.

### Neutrophil isolation and culture

Neutrophils were isolated from peripheral blood. Whole blood was mixed with 4 % dextran (Sigma-Aldrich) in 1 × phosphate buffered saline (PBS) and PBS at a ratio of 2:1:1 and the red blood cells were left to sediment on ice for 30 mins. The remaining supernatant was removed, layered over Ficoll-Paque (GE Healthcare, Buckinghamshire, UK) and centrifuged (400 g for 30 mins at room temperature). The remaining red bloods cells were lysed by addition of sterile molecular grade water (Sigma-Aldrich) and the suspension was quenched by the addition of PBS. The suspension was then washed (400 *g* for 10 mins at 4 °C) and the granulocyte number was assessed by trypan blue exclusion. Granulocytes were re-suspended at a density of 1 × 10^6^ per ml in supplemented RPMI-1640 media. The purity of the neutrophil preparation was approximately 95 % (confirmed by Rapi-diff staining as previously described [32]). Cytospin preparations were air dried, fixed with methanol for ten minutes and stained with Rapi-diff according to manufacturer’s instructions (Triangle, Skelmersdale, UK). A total of 200 cells per slide were counted and the proportion of neutrophils was calculated.

#### Neutrophil shape change, viability and CD11b and CD66b expression

Neutrophils were seeded at 5 × 10^5^ cells per polypropylene tube and incubated with ECVE (0.001–0.1 OD) for 2, 4 or 6 h (shape change and CD11b and CD66b expression) or 6 h (viability) in a 5 % CO_2_ humidified atmosphere at 37 °C. Cells were washed in PBS and prepared for flow cytometry. Details can be found in Additional file 3.

#### MMP-9 and CXCL8 release

Neutrophils were seeded at 1 × 10^5^ cells per well in a flat bottomed 96-well plate and incubated with ECVE, CSE (0.001–0.1 OD) or acrolein (Sigma-Aldrich) for 6 h in a 5 % CO_2_ humidified atmosphere at 37 °C. Supernatants were removed and analysed for MMP-9 and CXCL8 by enzyme linked immunosorbant assay (ELISA; R&D Systems, Abingdon, UK) according to manufacturer’s instructions. Supernatants were also used to measure MMP-9 activity by zymography and MMP-9 levels by western blot. Details can be found in Additional file 3.

For studies examining the effect of drug treatment on ECVE (0.003 OD) induced MMP-9 release, neutrophils were pre-treated with vehicle control (DMSO 0.005 %) the p38 MAPK inhibitor BIRB-796 (1 μM; Sigma-Aldrich), the ERK 1/2 inhibitor selumetinib (1 μM; Stratech Scientific Ltd, Newmarket, UK) or dexamethasone (1 μM; Sigma-Aldrich) for 1 h prior to incubation with ECVE. MMP-9 was measured by ELISA.

#### NE activity

Neutrophils were seeded at 1.4 × 10^5^ cells per well in a flat bottomed 96-well plate and incubated with ECVE (0.001–0.1 OD) for 6 h in a 5 % CO_2_ humidified atmosphere at 37 °C. A rhodamine 110-based substrate (R6506 Invitrogen) was added 30 min prior to the endpoint after which fluorescence was measured using a FLUOstar omega plate reader set at excitation 485 nm emission 520 nm (BMG Labtech, Buckinghamshire, UK).

#### Inflammatory pathway activation

Neutrophils were seeded at 1 × 10^6^ cells per well in a flat bottomed six-well plate and incubated with ECVE (0.003 and 0.01 OD) for 30 and 60 min in a 5 % CO_2_ humidified atmosphere at 37 °C. Cells were then lysed in RIPA buffer [10 mM Tris–HCl, pH 7.4, 150 mM NaCl, 1 mM EDTA, 1 % Nonidet P-40, 0.25 %] containing phosphatase (Sigma-Aldrich) and protease inhibitors (Calbiochem, Nottingham, UK) and samples were prepared for Western blot. Details can be found in Additional file 3.

### Ultra-high performance liquid chromatography mass spectrometry (UHPLC-MS) analysis

UHPLC-MS was performed to analyse the chemical composition of e-liquid and ECVE. Details can be found in Additional file 3.

### Data analysis

Statistical analyses were performed using GraphPad InStat software (GraphPad Software Inc., California, USA). Experiments with ECVE dose response curves were analysed using a one-way analysis of variance followed by a Dunnett’s post-test.

## Results

### Neutrophil activation

**Fig. 1:**
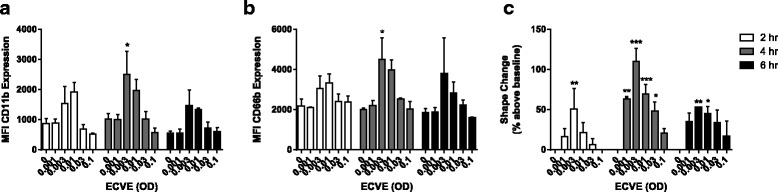
E-cig induced neutrophil activation. Neutrophils from three healthy subjects were exposed to ECVE (0.001–0.1 OD) for 2, 4 or 6 h (white, grey and black bars respectively). Neutrophils were analysed for CD11b (**a**) and CD66b (**b**) expression, and shape change (**c**) by flow cytometry. Data presented as mean ± SEM where *, ** and *** = significant increase compared to unstimulated control (*p* < 0.05, *p* < 0.01, and *p* < 0.001 respectively)

Neutrophils (*n* = 3) were exposed to ECVE for up to 6 h. ECVE increased neutrophil shape change and the mean fluorescence intensity of CD11b and CD66b expression compared to untreated neutrophils (Fig. 1 and Additional file 4); maximal changes were observed with 0.003 ECVE for 4 h (*p* < 0.05). There was a bell shaped curve, as shape change, CD11b and CD66b expression levels were not increased at higher ECVE concentrations. Analysis using the percentage of CD11b positive neutrophils followed a similar trend but this did not reach statistical significance (Additional file 5), while there was no change for the percentage of CD66b positive neutrophils (Additional file 5). There was high baseline expression of both of these markers when using percentage expression analysis. Following ECVE treatment for 6 h, the percentage of neutrophils undergoing early apoptosis or necrosis were not increased compared to untreated neutrophils (Additional file 6).

### Protease and CXCL8 secretion

**Fig. 2:**
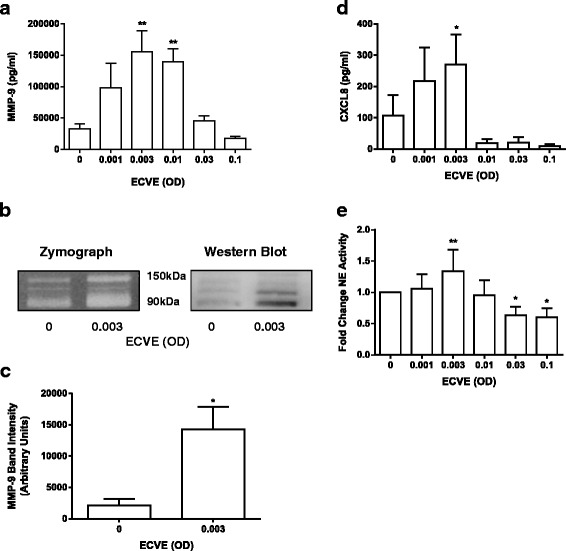
E-cig induced protease and CXCL8 release. Neutrophils from healthy subjects were exposed to ECVE (0.001–0.1 OD) for 6 h and supernatants were analysed for MMP-9 (**a**) and CXCL8 (**d**) release by ELISA (*n* = 10) and NE activity by florescence (**e**; *n* = 6). Culture supernatants from healthy subjects (0.003 ECVE) were also analysed for MMP-9 activity by zymography and MMP-9 expression by western blot (representative image; (**b**)). Western blots were analysed by densitometry (**c**). Data presented as mean ± SEM where *, ** and *** = significant change compared to unstimulated control (*p* < 0.05, *p* < 0.01, and *p* < 0.001 respectively)

Neutrophils (*n* = 10) exposed to ECVE secreted increased quantities of MMP-9 and CXCL8 with maximal release observed at 0.003 ECVE (*p* < 0.01 and *p* < 0.05 respectively; Fig. 2a and d). The secretion of MMP-9 and CXCL8 showed a bell shaped curve, with reduced secretion at higher concentrations. We confirmed that there was no contamination of the ECVE by the apparatus, by preparing a “mock” ECVE, bubbling air through the culture medium (Additional file 7).

Gelatinase activity in neutrophil supernatants (*n* = 3) was increased following exposure to 0.003 ECVE (Fig. 2b). Activity was observed in the 90–150 kDa range, consistent with different forms of MMP-9. Western blot analysis confirmed MMP-9 was present within the 90–150 kDa range (Fig. 2b), and densitometry confirmed a significant increase in MMP-9 secretion upon exposure to 0.003 ECVE compared to untreated neutrophils (Fig. 2c).

NE activity was significantly increased in the supernatants of neutrophils (*N* = 6) exposed to 0.003 ECVE compared to untreated neutrophils (*p* < 0.01; Fig. 2e). A bell shaped curve was again observed, with a significant reduction in NE activity at 0.03 and 0.1 ECVE (*p* < 0.05).

### Cell signalling pathway activation

Cigarette smoke has previously been shown to activate the p38 MAPK and ERK pathways in neutrophils [33–35]. In addition, nicotine has been shown to activate the NF-κB pathway ([36]. We therefore examined the effect of ECVE on the activation of these pathways.

**Fig. 3:**
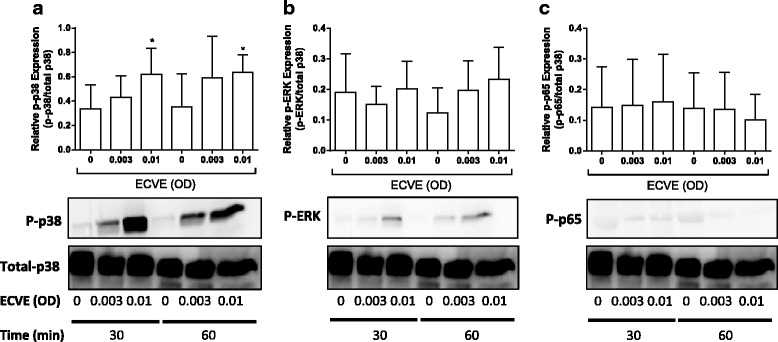
E-cig induced cell signalling pathway activation. Neutrophils from five healthy subjects were exposed to ECVE (0.003 and 0.01 OD) for 30 or 60 mins and cell lysates were analysed for **a** phospho-p38 MAPK, **b** phospho-ERK and **c** phospho-p65. All blots were analysed by densitometry and any changes were relative to total p38 MAPK. Data presented as mean ± SEM where * = significant increase compared to unstimulated control (*p* < 0.05)

Western blot analysis demonstrated a significant increase in p38 MAPK phosphorylation at 0.01 ECVE (*p* < 0.05) and a numerical increase at 0.003 compared to unstimulated controls (*n* = 5; Fig. 3). There was a numerical increase in ERK 1/2 phosphorylation that did not reach statistical significance. There were no changes in the phosphorylation of the p65 subunit of nuclear factor kappa-light-chain-enhancer of activated B cells (NF-κB).

**Fig. 4:**
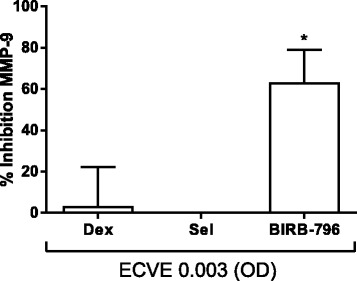
Inhibition of e-cig induced MMP-9 release. Neutrophils from six healthy subjects were pre-incubated with dexamethasone (dex; 1 μM), selumetinib (Sel; 1 μM) or BIRB-796 (1 μM) for 1 h prior to ECVE (0.003) exposure for 6 h. Supernatants were analysed for MMP-9 by ELISA. Data presented as mean ± SEM where * = significant increase compared to unstimulated control (*p* < 0.05)

We further investigated the involvement of p38 MAPK in ECVE induced neutrophil activation by studying the effect of a p38 MAPK inhibitor, BIRB-796, on MMP-9 release in response to ECVE (*n* = 6). We used 0.003 ECVE as this concentration caused the greatest secretion of MMP-9 (Fig. 2). Pre-treatment of neutrophils with BIRB-796 (1 μM) for 1 h prior to incubation with 0.003 ECVE for 6 h significantly reduced MMP-9 release by 62 % (Fig. 4). The ERK inhibitor, selumetinib (1 μM), and the corticosteroid dexamethasone (1 μM) had no effect.

### E-Cig brand and CSE comparisons

**Fig. 5:**
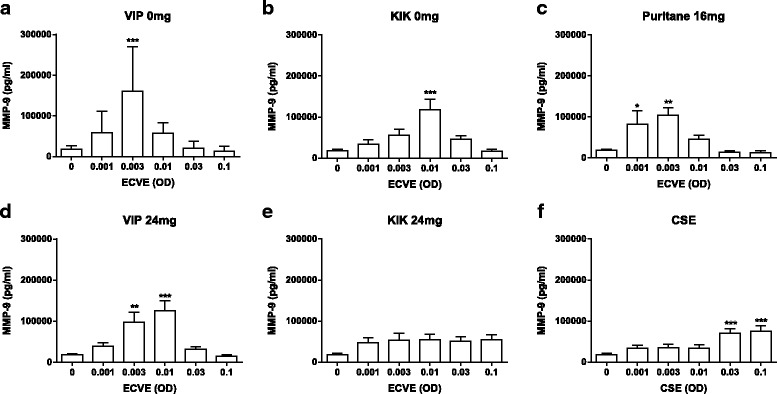
E-cig induced MMP-9 release; brand comparisons. Neutrophils from ten healthy subjects were exposed to brand 1 (0 or 24 mg; (**a** and **d**)), brand 2 (0 or 24 mg; (**b** and **e**)), brand 3 (16 mg; (**c**)), or CSE (**f**) for 6 h. Supernatants were analysed for MMP-9 by ELISA. Data presented as mean ± SEM where *, ** and *** = significant increase compared to unstimulated control (*p* < 0.05, *p* < 0.01, and *p* < 0.001 respectively)

**Fig. 6:**
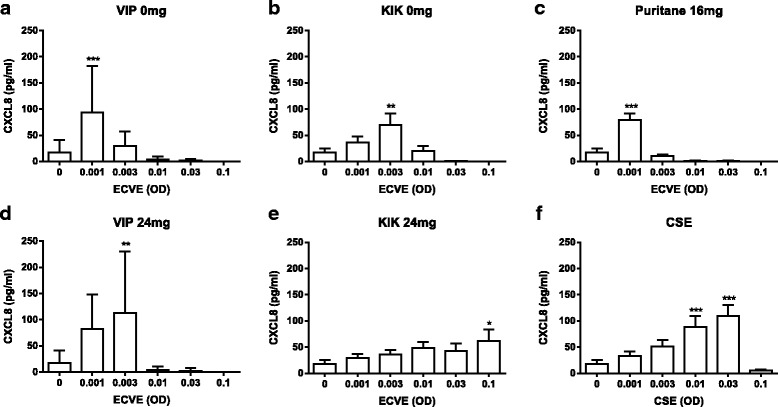
E-cig induced CXCL8 release; brand comparisons. Neutrophils from ten healthy subjects were exposed to brand 1 (0 or 24 mg; (**a** and **d**)), brand 2 (0 or 24 mg; (**b** and **e**)), brand 3 (16 mg; (**c**)), or CSE (**f**) for 6 h. Supernatants were analysed for CXCL8 by ELISA. Data presented as mean ± SEM where *, ** and *** = significant increase compared to unstimulated control (*p* < 0.05, *p* < 0.01, and *p* < 0.001 respectively)

Nicotine has previously been shown to induce CXCL8 release from neutrophils [36]. We therefore sought to evaluate the effects of different e-cig brands with and without nicotine on MMP-9 and CXCL8 release (*n* = 10; Figs. 5 and 6).

All the brands increased MMP-9 and CXCL8 secretion (although the increase in MMP-9 secretion after exposure to KIK 24 mg did not reach statistical significance), with bell shaped curves observed for most brands. The ECVE concentrations that caused the greatest inductions of MMP-9 and CXCL8 secretion varied greatly between brands. CSE caused significant increases in MMP-9 secretion at 0.03 and 0.1 OD and CXCL8 secretion at 0.01 and 0.03 OD. The effects of the different e-cig brands were often similar to, or greater than, CSE.

### UHPLC-MS analysis

The labelling stating the constituents of brand 1 (24 mg) e-cig liquid was as follows: nicotine, water, propylene glycol, glycerol and flavours. We investigated the constituents of e-cig liquid using UHPLC-MS. Chemicals were putatively identified based on molecular weight, with other constituents unidentified. Their chemical class and potential hazards are documented within Table 2, along with the associated chemical entities of biological interest (ChEBI) code [37], which enables cross-referencing of chemicals for biological relevance. Cyclohexane, isoquinoline, diglyme and skatole were identified.

**Table 2:** Chemicals putatively identified in brand 1 (24 mg) e-liquid

Chemical	Hazard statement	Hazard class	Type of compound	ChEBI code
Anabasine;nicotine;TC-2403-12;;	H301/H310/H400/H411	Toxic/Dangerous	Active drug	18723
Glycerol	N/A	N/A	Solubilizing agent	17754
1,4-Cyclohexanedione;2-hexenedial	S22/S23/S24/S25	Irritant	Cyclohexane analogue	28286; N/A
Skatole;tolylacetonitrile;;	H315/H319/H335/H411	Warning/Senatizer	Coal tar extract - flavour	9171; 27982
1-Methoxy-2-(2-methoxyethoxy)ethane	H226/H360FD	Health Hazard/Flammable	Diglyme - toxic digylcerol breakdown product	46784
Nicotyrine	N/A	Toxic/Dangerous	Nicotine breakdown product	7564
methyl 10,12-dihydroperoxy-8E,13E,15Z-octadecatrienoate	N/A	N/A	Fatty acid	N/A
Isoquinoline;quinoline;;	H302/H310/H315	Acutely Toxic/Dangerous	Bicyclic pyridine analogue	16092; 17362
8-hydroxy-nonanoic acid	N/A	N/A	Hydroxylated fatty acid	N/A
2,3,5-Trihydroxytoluene	N/A	N/A	N/A	17185
Monocyclic botryococcane;;	N/A	N/A	Isoprenyl-lipid	N/A
1-Methoxy-2-(2-methoxyethoxy)ethane;“2-methylpentane-1,2,4-triol”;;	H226/H360FD	Health Hazard/Flammable	Diglyme - toxic digylcerol	46784
2,3-Dihydroxyvaleric acid	N/A	N/A	Pentose-sugar	N/A
Pentaethylene glycol monodecyl ether;;	N/A	N/A	Detergent	N/A
Unknown lipid	N/A	N/A	N/A	N/A
Unknown lipid	N/A	N/A	N/A	N/A
Unknown lipid	N/A	N/A	Sphingolipid	N/A
Isopentenyladenine-7-N-glucoside;;12-oxo-20-trihydroxy-leukotriene B4;;	N/A	N/A	N/A	74321; N/A
Ethyl-trimethyl-silane	N/A	N/A	Potential silica breakdown product from wick	48788

A similar analysis was carried out on ECVE prepared using brand 1 (24 mg) e-cig liquid. Over 120 chemicals were putatively identified (Table 3), with extra constituents potentially coming from e-cig liquid vaporisation by-products. Known harmful chemicals including acrolein, propanal, styrene and the carcinogens 2, 3-benzofuran and allylthiourea were putatively identified.

**Table 3:** Chemicals putatively identified in ECVE prepared with brand 1 (24 mg)

Chemical	Hazard statement	Hazard class	Type of compound	ChEBI
Nicotine	H301/H310/H400/H411	Toxic/Dangerous	Active drug	18723
Glycerol	N/A	N/A	Solubilizing agent	17754
gamma-hexenoic acid	H314/R34	Danger/Corrosive	Unsaturated fatty acid	38355
2-methyl valeric acid	H314/R34	Danger/Corrosive	Saturated fatty acid	N/A
2-Methylpentane-1,2,4-Triol	N/A	N/A	Saturated polyol	N/A
4-Aminohydrocinnamic Acid	H315/H319/H335	Warning/Irritant	saturated carboxylic acid	N/A
5-oxo-7-octenoic acid	N/A	N/A	Branched fatty acid	N/A
Piperidine	H225/H311/H331/H314	Corrosive/Highly Flammable/Toxic	Organic Heterocyclic	18049
Propanal	H225/H302/H332/H315/H318/H335	Highly Flammable/Harmful/Irritant	Small organic (possible glycerol breakdown)	17153
(2R,3S)-2,3-Dimethylmalate	N/A	Natural	Possible cellular metabolite	57422
2,3-Benzofuran	H226/H351	Flammable/Carcinogen	Heterocyclic	36790
4-(3-pyridyl)-butanoate	N/A	N/A	Nitrosonornicotine metabolite derived from tobacco smoke.	66942
N-Nitroso-1,3-thiazolidine	N/A	N/A	Polar small molecule	7329
allylthiourea	H301	Toxic/Carcinogen	Polar small molecule	74079
2-methyl-1-butanol	N/A	N/A	Polar small molecule	50625
Acrolein	H225/H301/H311/H314/H330/H400	Poisonous/Irritant/Flammable	Glycerol breakdown product	15368
Styrene	H226/H315/H319/H332	Harmful/Irritant	Unsaturated aromatic	27452

We also performed pure standard analysis of acrolein (Additional file 8). Inference of median ECVE ion count levels of acrolein from the standard analysis indicates a level of 4.81 μM present in ECVE.

### Effect of acrolein on neutrophil response

**Fig. 7:**
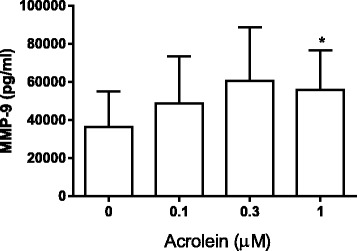
Acrolein induced MMP-9 release. Neutrophils from six healthy subjects were exposed to acrolein (0.1–1 μM) for 6 h. Supernatants were analysed for MMP-9 by ELISA. Data presented as mean ± SEM where * = significant increase compared to unstimulated control (*p* < 0.05)

The presence of high acrolein levels (4.81 μM) in ECVE prepared with brand 1 (24 mg) led us to examine the effects of acrolein (0.1–1 μM) on the neutrophil inflammatory response from six healthy subjects. Acrolein caused a significant increase in MMP-9 release at a concentration of 1 μM (Fig. 7).

## Discussion

ECVE causes activation of human neutrophils. ECVE increased neutrophil CD11b and CD66b expression, and induced neutrophil shape change; these are markers of neutrophil activation. Furthermore, ECVE increased MMP-9 and CXCL8 secretion by neutrophils, and upregulated MMP-9 and NE activity. These pro-inflammatory changes were accompanied by p38 MAPK activation. Mass spectroscopy analysis of e-cig liquid and ECVE confirmed the presence of various compounds capable of causing harm to humans. These findings challenge the perception that e-cigs are safe.

It is known that acute exposure to cigarette smoke extract increases CXCL8 and MMP-9 secretion from alveolar macrophages and neutrophils, [7, 38] and NE secretion from neutrophils [8, 16]. Here we show similar findings for the effects of ECVE on neutrophils. Neutrophil activation is a characteristic feature of COPD; indeed, COPD is often referred to as a “neutrophilic lung disease”. We demonstrate that e-cigs cause neutrophil activation, which raises concerns about the safety of these devices.

A proposed advantage of e-cigs is that they lack the carcinogens within the tar present in cigarettes. However, e-cigs may still contain compounds with carcinogenic properties [5]. We now show that e-cigs also contain a variety of other compounds, including carcinogens. These include the saturated aldehyde propanal, the carcinogens 2, 3-benzofuran and allylthiourea and the respiratory toxin styrene.

We observed acrolein in the vapour extract but not the e-liquid itself. Acrolein can be produced by heating glycerol [39], and our observations are likely attributable to heating glycerol contained in e-liquid. Acrolein is also a constituent of tobacco smoke. Acrolein induces inflammatory responses from macrophages and epithelial cells [40, 41]. We now show that acrolein induces MMP-9 release from neutrophils within the concentration found in ECVE. This confirms that acrolein is one potential cause of the pro-inflammatory effects of e-cig vapour. We have identified a number of chemicals present in ECVE using UHPLC-MS, and the potential for each of these to cause inflammation or other toxic effects can now be investigated. Styrene has previously been linked to airway inflammation present in people with occupational asthma [42] and has also been shown to induce monocyte chemotactic protein-1 (MCP-1) and CXCL8 release from an alveolar epithelial cell line [43, 44]. In addition, propanal has been shown to increase gene expression of several pro-inflammatory cytokines and chemokines including IL-6 and CXCL8 in an alveolar epithelial cell line [45].

We observed a bell shaped curve for the effect of ECVE in all experiments of neutrophil function. This was not due to a reduction in neutrophil viability. There are other examples of bell shaped curves for neutrophil biology, including CXCL8 induced chemotaxis, substance P induced adhesion and *Staphylococcus aureus* induced CD11b, CD18 and CD66b expression [46–48]. It has been suggested that the bell shaped curve for neutrophil function arises due to negative feedback mechanisms. For example, CXCL8 causes a dose dependant reduction in chemokine C-X-C motif receptor (CXCR) 1 and CXCR2 expression. Reducing the expression of these chemotactic receptors regulates neutrophil inflammatory responses and arrests these cells at the site of inflammation where chemokine concentrations are highest.

CD66b cross-linking between neutrophils induces CXCL8 release [49]. When neutrophils are exposed to ECVE, CD66b expression may be driving the release of inflammatory mediators through neutrophil cross-linkage. However, at higher ECVE concentrations this CD66b response diminishes. This may regulate neutrophil responses to avoid excessive inflammation, similar to the suggestion of Schmidt *et al* in the case of neutrophils exposed to bacteria [47]. However, neutrophils artificially arrested in this state are less likely to exert their protective innate immune functions and therefore the host may be more susceptible to bacterial infection.

The analysis of CD11b and CD66b principally used median fluorescence intensity (MFI) by flow cytometry to provide a sensitive overall quantification of expression in all neutrophils. The analysis of cell percentages is a commonly used alternative, but in this case there were high baseline expression levels which made it difficult to observe any increase with ECVE.

MMP-9 levels are increased in the lungs of smokers [50] and there is a positive correlation between MMP-9 levels, sputum neutrophils and COPD severity [17, 18, 51]. Similarly, NE levels are increased in the lungs of cigarette smokers and NE activity is positively correlated with COPD severity [19, 52]. MMP-9 and NE contribute to emphysema through the destruction of the lung parenchyma. Our results suggest that e-cig use increases the ability of human neutrophils to secrete activated MMP-9 and NE, which may contribute to lung parenchymal destruction.

NE degrades the antimicrobial peptide, short palate lung and nasal epithelium clone 1 (SPLUNC1) [53]. E-liquid inhibits SPLUNC1 expression in human airway epithelial cells [10]. Both direct and indirect reductions in SPLUNC1 levels by e-cigs in addition to reduced bacterial phagocytic activity of e-cig exposed alveolar macrophages [54], may result in a susceptibility to bacterial infections in e-cig users. Mice exposed to e-cig vapour have been shown to be more susceptible to viral and bacterial infections [54]. This is reminiscent of the effects of tobacco smoking.

ECVE caused activation of the p38 MAPK pathway, akin to tobacco smoke exposed alveolar macrophages [7, 41]. P38 MAPK activation promotes inflammation and activated p38 MAPK is increased in COPD lungs [52, 55]. We showed activation of the p38 MAPK pathway at 0.01 ECVE. The results at 0.003 ECVE were not statistically significant, despite clear activation in some of the experiments performed (Fig. 3). The 0.003 ECVE concentration generally caused the greatest increases in cell activation (Figs. 1 and 2), although there was evidence that 0.01 ECVE caused significant MMP-9 release and neutrophil shape change. The p38 MAPK inhibitor BIRB-796 suppressed 0.003 ECVE induced MMP-9 release by approximately 60 %, confirming the role of p38 MAPK signalling in the neutrophil response to ECVE. However, approximately 40 % of ECVE induced MMP-9 release was insensitive to p38 MAPK inhibition suggesting ECVE can also induce MMP-9 release by p38 MAPK independent mechanisms.

The corticosteroid dexamethasone had no effect on ECVE induced MMP-9 release. This may be relevant to COPD patients using e-cigs and corticosteroids; these drugs do not appear to suppress neutrophil activation caused by e-cigs.

E-cig induced MMP-9 and CXCL8 release was demonstrated using different brands of e-cigs with and without nicotine. This demonstrates that the pro-inflammatory effects are not restricted to one brand and are observed using preparations with and without nicotine. There were considerable differences in the magnitude of effects between the brands, and the shape of the dose response curves, which are likely attributable to differences in chemical composition. The increased flow rate used to produce ECVE with brand 3 did not result in an increased magnitude of effect when compared to the other brands. Our comparisons with cigarette smoke provide important insights; MMP-9 and CXCL8 release caused by different e-cig brands were often similar to, or in excess of, the CSE response.

Nicotine has previously been shown to induce CXCL8 release from neutrophils [36]. We observed increased release of MMP-9 and CXCL8 upon exposure to ECVE with and without nicotine, suggesting that these effects are dependent on other pro-inflammatory constituents.

The primary aim of this paper was to examine the potential of e-cigs to cause harm. We used neutrophils from healthy never smokers to avoid any confounding variables due to chronic exposure to tobacco cigarettes or the presence of chronic inflammation. Nevertheless, future studies should examine the effect of e-cigs on immune cells isolated from COPD patients, particularly those involved in the pathogenesis of COPD, including neutrophils, alveolar macrophages and CD8 T-cells. This will help us to understand the impact of e-cigs on patients with established chronic inflammation.

ECVE causes rapid activation of neutrophils which continues up to 6 h. Although we do not know how repeated exposure to e-cigs will affect neutrophils, the acute effects reported here are similar to those by cigarette smoke extract. This suggests that chronic exposure to e-cigs may induce similar chronic inflammatory changes reported in cigarette smokers.

## Conclusions

We have shown that ECVE causes pro-inflammatory responses from human neutrophils. E-cigs are often used to avoid the unwanted effects of conventional cigarettes, such as causing pulmonary inflammation. Our results raise concerns over the safety of e-cig use.

## Additional files

Additional file 1: Demographics of the study population. (DOCX 13 kb) Additional file 2: The absorbance spectrum ECVE preparations. The optical density of e-cig vapour extract preparations for VIP 0 mg (blue), VIP 24 mg (red), KIK 0 mg (green), KIK 24 mg (yellow), Puritane (orange) and cigarette smoke extract (CSE; pink) were measured from 200–1000 nM. (PPTX 93 kb) Additional file 3: Supplementary methods. (DOCX 28 kb) Additional file 4: Raw flow cytometry data. Flow cytometry plots of ECVE exposed neutrophils examined for CD11b (left panel), CD66b (middle panel) and shape change (right panel). The light grey peaks indicate the unstained control and the dark grey peaks indicate the antibody staining. (PPTX 274 kb) Additional file 5: The percentage of CD11b and CD66b positive neutrophils. Neutrophils from three healthy subjects were exposed to ECVE (0.001–0.1 OD) for 2, 4 or 6 h (white, grey and black bars respectively). Neutrophils were analysed for CD11b (a) and CD66b (b) expression by flow cytometry. Data presented as mean ± SEM (PPTX 178 kb) Additional file 6: Effect of e-cig exposure on neutrophil viability. Neutrophils from three healthy subjects were exposed to ECVE (0.001–0.1 OD) for 6 h and the percentage of cells undergoing early apoptosis (a) or late apoptosis (b) was quantified by flow cytometry. Data presented as mean ± SEM. (PPTX 133 kb) Additional file 7: A comparison between a mock ECVE preparation and media alone. Neutrophils from 10 healthy subjects were exposed to a mock ECVE preparation or media alone for 6 h and supernatants were analysed for MMP-9 release by ELISA. (PPTX 62 kb) Additional file 8: The concentration of selected harmful chemicals contained within ECVE. The concentration of acrolein was estimated based upon the median ion count levels calculated from pure standard analysis. (PPTX 46 kb)
