# Supplementary material for: Electronic cigarette exposure triggers neutrophil inflammatory responses
Source: Respir Res. 2016 May 17;17:56. doi: 10.1186/s12931-016-0368-x (PMC4869345; doi:10.1186/s12931-016-0368-x)
Supplement: Additional file 1: — Demographics of the study population. (DOCX 13 kb) [file 12931_2016_368_MOESM1_ESM.docx]

|  | **Healthy Subjects** |
| --- | --- |
| **n** | 10 |
| **Age** | 35.5 (9.8) |
| **Sex (M/F)** | 5/5 |
| **FEV_1_ (L)** | 3.6 (0.7) |
| **FEV_1_ % Predicted** | 100.6 (11.7) |
| **FVC (L)** | 4.6 (1) |
| **FEV_1_/FVC Ratio (%)** | 78.9 (6.0) |
| **Pack Year History** | 0 |

**Additional File 1. Demographics of the study population.** Data shown are mean (sd). FEV1: forced expiratory volume in 1 s, FVC: forced vital capacity.
