# Supplementary figures and images for: Electronic cigarette exposure triggers neutrophil inflammatory responses
Source: Respir Res. 2016 May 17;17:56. doi: 10.1186/s12931-016-0368-x (PMC4869345; doi:10.1186/s12931-016-0368-x)

## Slide 1
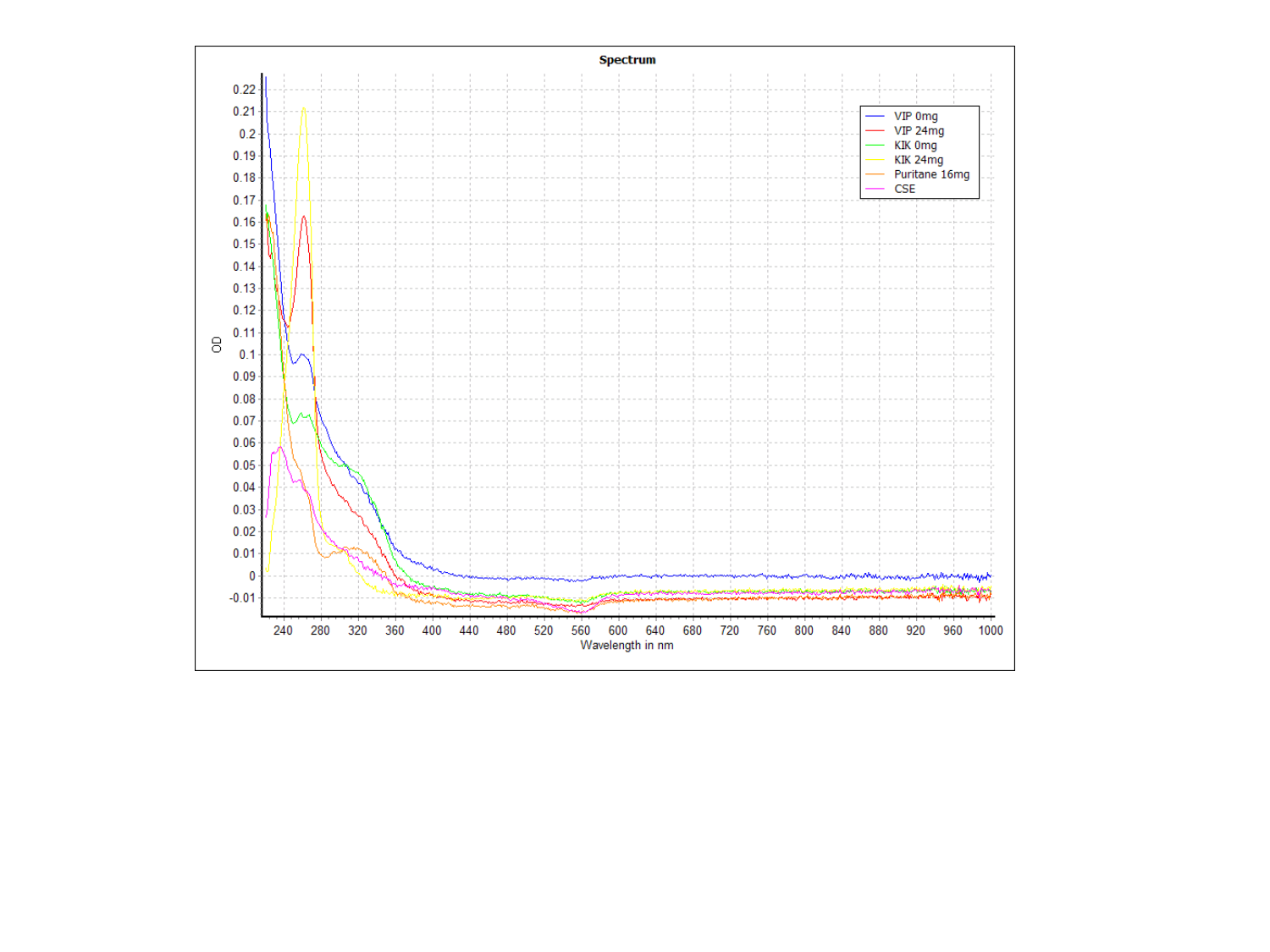

Supplement: Additional file 2: — The absorbance spectrum ECVE preparations. The optical density of e-cig vapour extract preparations for VIP 0 mg (blue), VIP 24 mg (red), KIK 0 mg (green), KIK 24 mg (yellow), Puritane (orange) and cigarette smoke extract (CSE; pink) were measured from 200–1000 nM. (PPTX 93 kb) [file 12931_2016_368_MOESM2_ESM.pptx]

## Slide 1
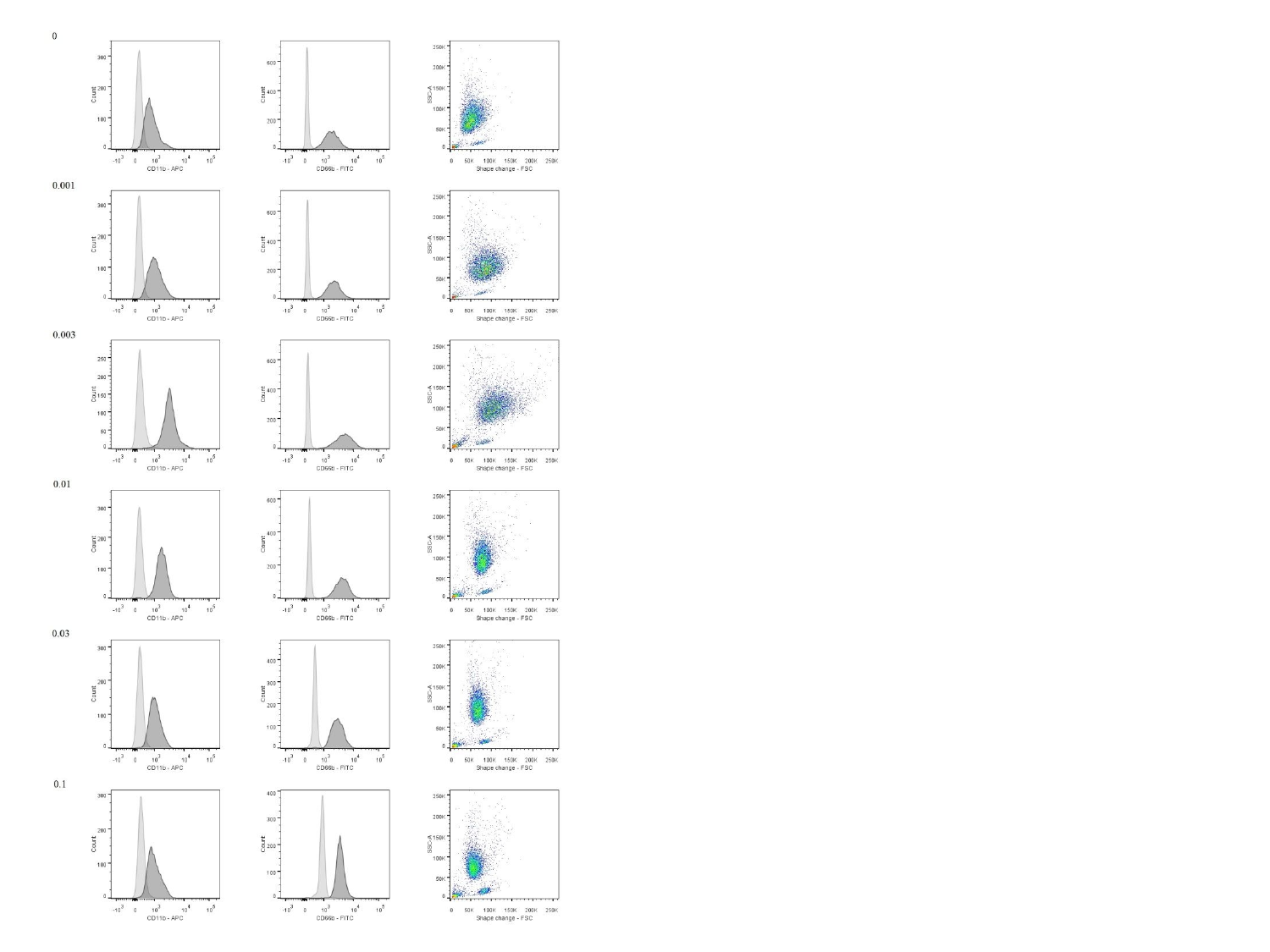

Supplement: Additional file 4: — Raw flow cytometry data. Flow cytometry plots of ECVE exposed neutrophils examined for CD11b (left panel), CD66b (middle panel) and shape change (right panel). The light grey peaks indicate the unstained control and the dark grey peaks indicate the antibody staining. (PPTX 274 kb) [file 12931_2016_368_MOESM4_ESM.pptx]

## Slide 1
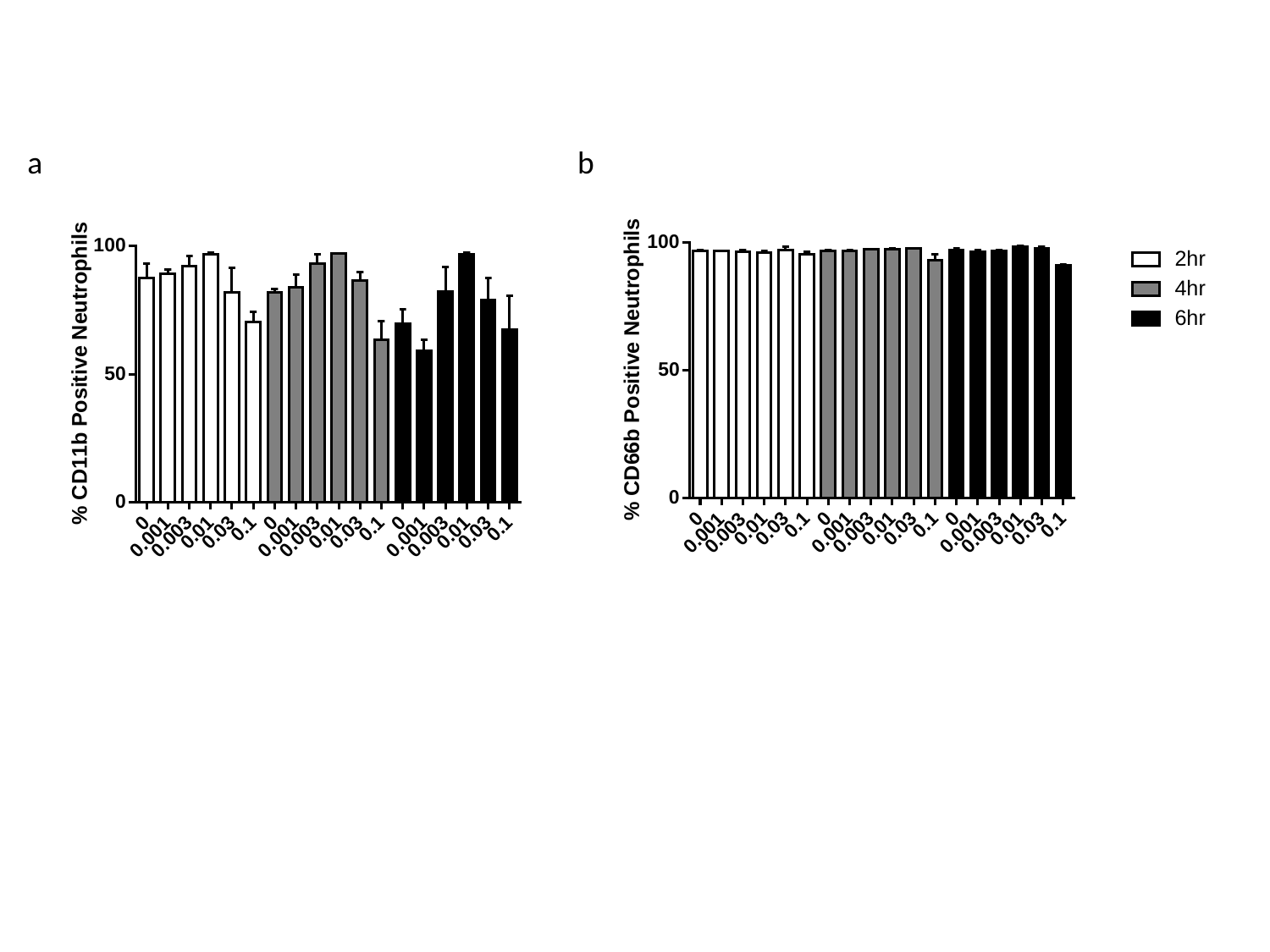

a
b

Supplement: Additional file 5: — The percentage of CD11b and CD66b positive neutrophils. Neutrophils from three healthy subjects were exposed to ECVE (0.001–0.1 OD) for 2, 4 or 6 h (white, grey and black bars respectively). Neutrophils were analysed for CD11b (a) and CD66b (b) expression by flow cytometry. Data presented as mean ± SEM (PPTX 178 kb) [file 12931_2016_368_MOESM5_ESM.pptx]

## Slide 1
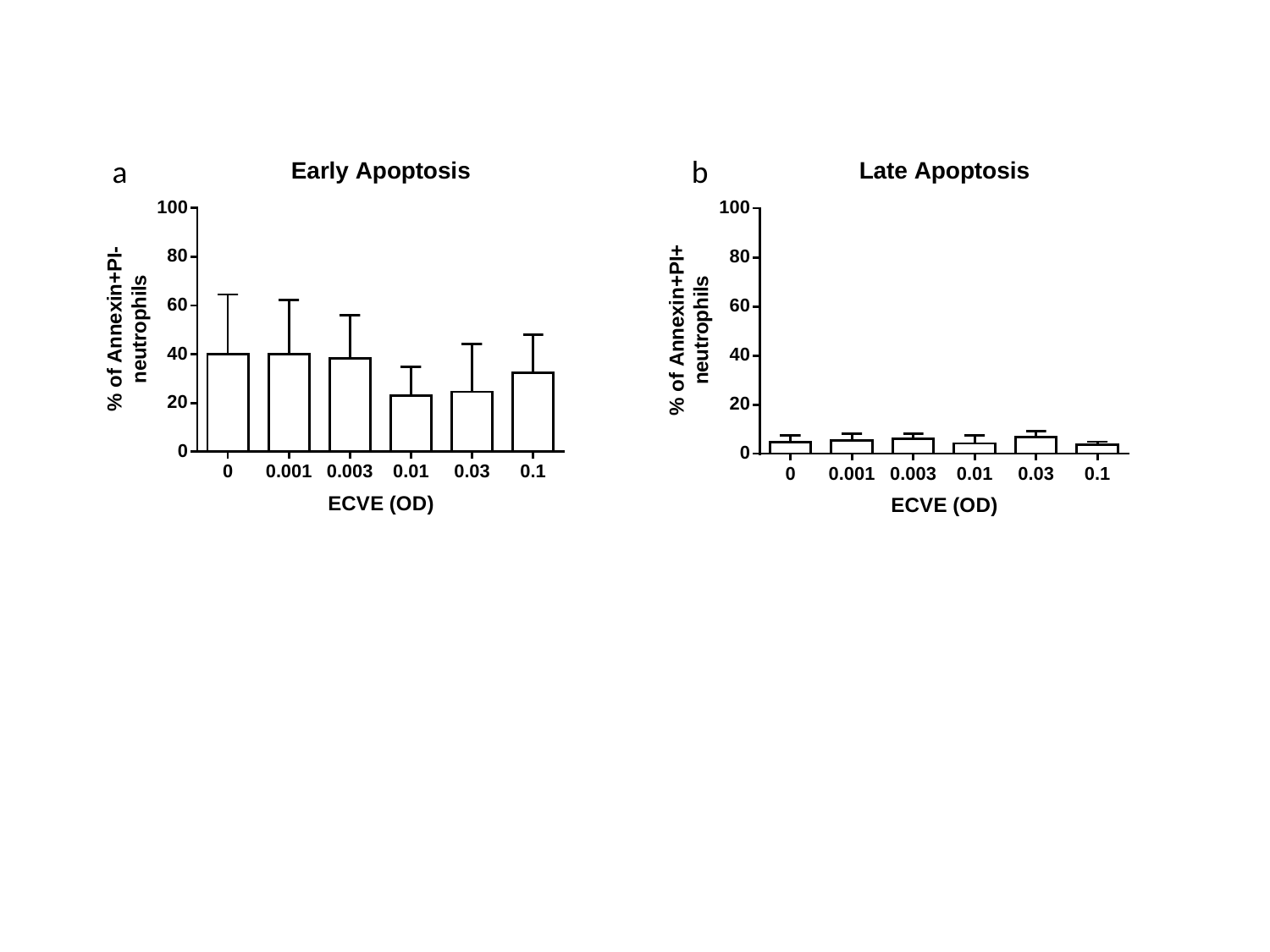

a
b

Supplement: Additional file 6: — Effect of e-cig exposure on neutrophil viability. Neutrophils from three healthy subjects were exposed to ECVE (0.001–0.1 OD) for 6 h and the percentage of cells undergoing early apoptosis (a) or late apoptosis (b) was quantified by flow cytometry. Data presented as mean ± SEM. (PPTX 133 kb) [file 12931_2016_368_MOESM6_ESM.pptx]

## Slide 1
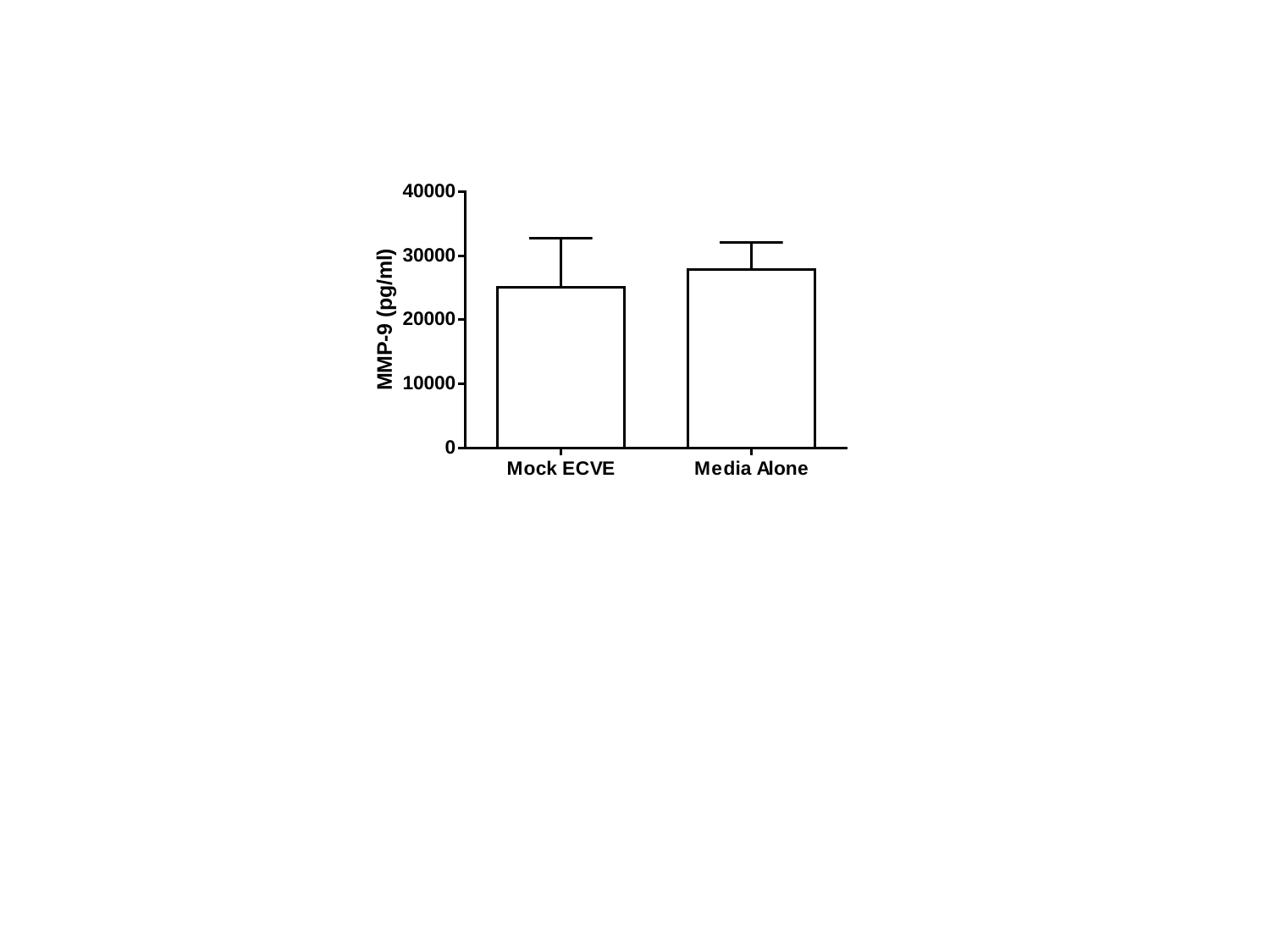

Supplement: Additional file 7: — A comparison between a mock ECVE preparation and media alone. Neutrophils from 10 healthy subjects were exposed to a mock ECVE preparation or media alone for 6 h and supernatants were analysed for MMP-9 release by ELISA. (PPTX 62 kb) [file 12931_2016_368_MOESM7_ESM.pptx]

## Slide 1
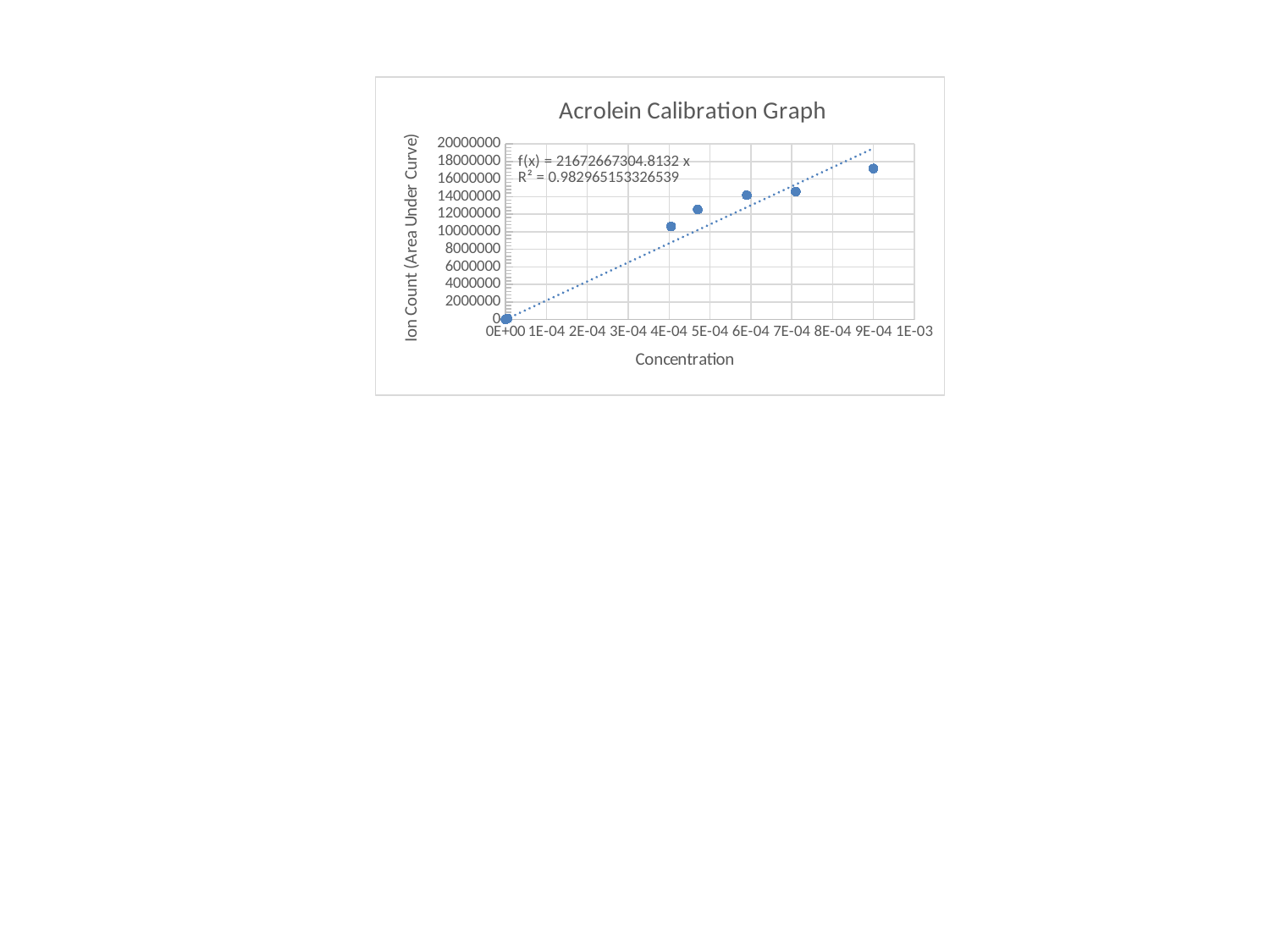

### Chart: Acrolein Calibration Graph
| Category | |
|---|---|

Supplement: Additional file 8: — The concentration of selected harmful chemicals contained within ECVE. The concentration of acrolein was estimated based upon the median ion count levels calculated from pure standard analysis. (PPTX 46 kb) [file 12931_2016_368_MOESM8_ESM.pptx]
