# Supplementary material for: Electronic cigarette exposure triggers neutrophil inflammatory responses
Source: Respir Res. 2016 May 17;17:56. doi: 10.1186/s12931-016-0368-x (PMC4869345; doi:10.1186/s12931-016-0368-x)
Supplement: Additional file 3: — Supplementary methods. (DOCX 28 kb) [file 12931_2016_368_MOESM3_ESM.docx]

**Flow Cytometry**

**CD11b, CD66b Expression and Neutrophil Shape Change**

Neutophils either remained unstained or were stained with specific monoclonal antibodies or the corresponding isotype control. The following antibodies were used: CD11b ICRF44- APC (isotype: mouse IgG1κ - APC); and CD66b G10F5- FITC (isotype: mouse IgMκ - FITC; all BD Biosciences, Oxford UK). Forward scatter/side scatter (FSC/SSC) plots were used to gate neutrophils from debris (FACSCanto II, BD Biosciences). Histograms were used to analyse CD11b and CD66b expression. Data was analysed using FlowJo Vx (FlowJo LLC, Oregon, US) softwareand expressed as mean fluorescence intensity (MFI), which was calculated based on the fluorescence values (mean channel) of the cells stained with antibody minus the fluorescence values for the respective isotype control, or percentage of positive cells. Unstained neutrophils were distinguished in FSC plots and the mean FSC value was used as a measure of shape change.

**Viability**

Neutrophils were stained with FITC Annexin V and propidium iodide staining solution (PI) using the fluorescein isothiocyanate (FITC) Annexin V Apoptosis Detection Kit (BD Biosciences), according to the manufacturer’s instructions.

**Zymography**

Cell supernatants were diluted in sample buffer [0.125 M Tris–HCl, pH 6.8, 4% SDS, 20% glycerol, and 0.04% bromophenol blue] and electrophoresed on SDS polyacrylamide gels (8%) containing 0.1% gelatin. The gels were washed [2.5% Triton X-100 in 0.05 M Tris–HCl pH 8.0, and 5 mM CaCl_2_] at room temperature and incubated overnight at 37^o^C in development solution [0.05 M Tris–HCl pH 8.0, and 5 mM CaCl_2_]. The following day gels were stained [0.1% Coomasie Brilliant Blue, methanol:acetic acid:water (4.5:1:4.5, v:v:v)] for 4 hours followed by incubation with a de-staining solution [methanol:acetic acid:water (4.5:1:4.5, v:v:v)] until gelatinase activity could be visualised as clear bands against a dark blue background.

**Western Blot**

Cell lysates diluted in sample buffer [62.5 mM Tris, 10% glycerol, 1% SDS, 1% β-mercaptoethanol, and 0.01% bromphenol blue, pH 6.8], were electrophoresed on SDS polyacrylamide gels (10%) and transferred to Hy-bond ECL membranes (Whatman International Ltd, Kent, UK). Membranes were incubated with blocking buffer [5% dried milk in Tris buffered saline containing 0.1% Tween 20 (TBS/Tween 20)] for 1 h at room temperature and then incubated with primary antibodies (diluted in blocking buffer) at 4 °C overnight (rabbit anti-phospho-p38 MAPK [Thr180/Tyr182], rabbit anti-phospho-NF-κB p65 [Ser536], rabbit anti-phospho-ERK 1/2, rabbit anti-total p38 MAPK ; Cell Signalling, Hertfordshire, UK). After washing in TBS/Tween 20, the membranes were incubated for 60 min with a peroxidase-conjugated secondary antibody (diluted in wash buffer) (horseradish peroxidase-conjugated goat anti-rabbit, Cell Signalling), washed again, and the antibody labelled proteins were visualized by enhanced chemiluminescence (Amersham Biosciences, Buckinghamshire, UK).

Cell supernatants were also analysed by western blot to confirm the presence of MMP-9 immunostaining. Briefly supernatants were diluted in sample buffer [0.125 M Tris–HCl, pH 6.8, 4% SDS, 20% glycerol, and 0.04% bromophenol blue] and western blot was completed as above. Membranes were probed with rabbit anti-MMP-9 (Cell Signalling).The following antibodies were used: rabbit anti-phospho-p38 MAPK [Thr180/Tyr182], rabbit anti-phospho-NF-κB p65 [Ser536], rabbit anti-phospho-ERK 1/2, rabbit anti-total p38 MAPK primary antibodies and horseradish peroxidase-conjugated goat anti-rabbit secondary antibody (Cell Signalling, Hertfordshire).

**Ultra-High Performance Liquid Chromatography Mass Spectrometry (UHPLC-MS) Analysis**

All solvents were of Optima grade and purchased from Sigma–Aldrich (Gillingham, UK) along with acrolein and allylthiourea pure chemical standards. HPLC grade formic acid was purchased from Fisher Scientific (Loughborough, UK). Mass spectrometer calibration solution and chromatography columns were purchased from Thermo-Fisher Scientific (Hemel Hempstead, UK).

For sample preparation of pure e-cig liquid, 10µL was dissolved in 100µL of 80/20 mixture of water/acetonitrile and then added to a 300µL fixed insert glass vial. Cell media infused with e-cig vapour was diluted 50/50 in a 80/20 mixture of water/acetonitrile and then added to a 300µL fixed insert glass vial and both type of sample were immediately taken for UHPLC-MS analysis. For sample preparation of chemical standards, 1M solutions in appropriate mobile phase were prepared and subsequent linear-series dilutions to appropriate values was carried out. Samples were then added to a 300µL fixed insert glass vial prior to analysis.

All UHPLC-MS work was carried out on a Thermo-Finnigan Orbitrap-LTQ XL™ hybrid mass spectrometer operated in positive ionization mode coupled to a Thermo Accela autosampler (Fisher Scientific, Bremen, Germany). This system was operated using Thermo Xcaibur and Thermo Tune Plus software. Upon injection of 10µL of each sample, chromatographic separations were performed on a Thermo Hypersil Gold 2.1µm C_18_ column at a solvent flow-rate of 400 µL min^−1^. Profiling analysis was carried out on the column was eluted with 0.1% formic acid in water (A) and 0.1% formic acid in acetonitrile (B). Gradient elution was initiated with 5% (B) for 5 min and subsequently ramped to 95% (B) over 15 min, followed by a 5 min isocratic elution at 95% (B) before a return to 95% (A) held for further 5 min for column equilibration. All samples were maintained at 4 °C within the autosampler refrigerator whilst the column was maintained at 50°C within the autosampler oven. Mass calibration was carried out in accordance with the manufacturer’s guidelines

Acquisition settings for initial profiling were carried out at 30,000 resolution in centroid and ran at 1 µ-scan per 400ms in the 100-1000 *m/z* range with source gasses set at Sheath Gas = 40 arb units, Aux Gas =0 arb units, Sweep Gas = 5 (all systems specific arbitrary units). The ESI source voltage was set to 3.8V, and capillary ion transfer tube temperature set at 275°C.

Raw e-cig data was then subsequently converted in to the netCDF data format using the file converter function within Xcalibur and then deconvolved using the XCMS algorithm within the R-studio statistical software package. This data matrix was then used as the input for a correlation mass-match identification from our in-house PutMedID library alongside the HMDB, LipidMaps and Sieve high-resolution mass spectral libraries.

Chemical standard data (area under curve) was plotted against associated concentration to determine linearity of response. The slope equation of these lines alongside e-cig area under curve values were then used to determine unknown concentrations values contained within the e-cig liquids.

**References**

1. Singh D, Edwards L, Tal-Singer R, Rennard S. Sputum neutrophils as a biomarker in COPD: findings from the ECLIPSE study. *Respiratory research.* 2010;11:77.

2. Metcalfe HJ, Lea S, Hughes D, Khalaf R, Abbott-Banner K, Singh D. Effects of cigarette smoke on Toll-like receptor (TLR) activation of chronic obstructive pulmonary disease (COPD) macrophages. *Clinical and experimental immunology.* 2014;176(3):461-472.
